# Women workers’ labour market engagement and financial autonomy: the mediating role of government initiatives in Kerala, India

**DOI:** 10.3389/fsoc.2026.1882363

**Published:** 2026-10-01

**Authors:** Shahbaz Babar Khan, P. Mubarak Rahman, Jasvinder Sidhu, Najul Laskar

**Affiliations:** 1 SRM University AP, Amaravati, India; 2 PMA SAFI School of Management, SAFI Institute of Advanced Study (Autonomous), Calicut, Kerala, India; 3 Federation University Australia, Ballarat, VIC, Australia; 4 Indira Gandhi National Open University, New Delhi, India

**Keywords:** empowerment theory, financial autonomy, government initiatives, Kerala economy, women’s labour market engagement

## Abstract

This research examines the interrelationship between the labour market engagement and financial autonomy of employed women, with a focus on the mediating role of government initiatives in Kerala, India. Despite high literacy rates and progressive social indicators in Kerala, low female labour force participation points to deep-seated socio-cultural and institutional barriers. Drawing on Human Capital Theory, the Gender and Development Framework, and Empowerment Theory, the study constructs a comprehensive model incorporating cultural, economic, and policy determinants. Because the sample comprises women who are already employed, the study examines *labour market engagement*—employed women’s active involvement in work, confidence in employability, access to job opportunities, and perceived professional growth—rather than labour force participation in its conventional sense. Using survey data collected from 1,357 women workers across five districts of Kerala and analyzed through Structural Equation Modelling in AMOS 28.0, the results indicate that cultural determinants are more strongly associated with women’s labour market engagement than economic factors alone. Labour market engagement is positively and significantly associated with financial autonomy, suggesting that economic engagement is linked to greater financial decision-making power and financial control among women. Bootstrapped mediation analysis further indicates that government initiatives such as Kudumbashree and the Women Entrepreneurship Mission partially mediate the association between labour market engagement and financial autonomy. Given the cross-sectional design, these findings represent empirical associations rather than causal effects. The study contributes to gendered labour economics by suggesting that employment needs to be accompanied by enabling institutional mechanisms for sustainable empowerment and equitable growth.

## 1 Introduction

Women’s participation in the labour market keeps acting as a fundamental determinant of inclusive growth, social equity, and sustainable development. Despite progressive legal frameworks and policy reforms in this regard, the gender gap in labour force participation has continued to persist globally. According to estimates, the global female LFP rate was 48.7 percent in 2023 compared with 73 percent for men, according to the International Labour Organization (2024), thus revealing the existence of structural and cultural barriers that continue to limit economic agency among women (International Labour Organization, 2024). Modern works validate the view that greater female participation is beneficial for productivity, household welfare, and intergenerational human capital formation (Duflo, 2012; Elborgh-Woytek et al., 2013; International Labour Organization, 2023). Beyond reinforcing macroeconomic resilience, improved economic participation contributes to social inclusion and the attainment of Sustainable Development Goals—in particular, SDGs 5 and 8 (UN Women, 2023). However, participation alone guarantees neither rights nor empowerment without equal pay, decent work, and financial autonomy—meaning the power to control one’s income, savings, and related financial decisions (Kabeer, 2021; Doss, 2023).

In the Indian context, female labour force participation reflects a paradoxical pattern of progress and decline. Despite steady improvements in female education and economic growth, women’s LFP fell from 32 percent in 2005 to 23 percent in 2019, with only a modest recovery to 37 percent in 2022–23 (Press Information Bureau, 2024). The studies of Goldin (1995) and Mammen and Paxson (2000) posit that women’s participation declines during early industrialisation and rises later as education and services expand. Yet, in India, much of the recent increase stems from low-wage self-employment and informal work rather than from quality, formal sector jobs (Economic Advisory Council to the Prime Minister, 2024; World Bank, 2023). Gender wage gaps, occupational segregation, and caregiving responsibilities continue to constrain women’s economic inclusion (Jayachandran, 2021). The labour market remains deeply segmented, reflecting both socio-cultural norms and institutional gaps in care infrastructure, transport safety, and workplace flexibility (World Bank, 2023).

Kerala offers a peculiar socio-economic paradox: even with near-universal literacy (93 percent) and leading social development indicators, the state’s female LFP falls below the national average, fluctuating between 25 and 30 percent (Ray Hajong and Kakarlapudi, 2024). Such a “Kerala paradox” suggests that attainment of higher education and social advancement alone cannot ensure women’s economic participation. Even as recent studies indicate marginal improvements among women with postgraduate education and regular wage employment, structural obstacles—traditional gender roles, lack of flexible working opportunities, and limited industrial diversification—remain binding constraints. Kerala’s relatively progressive policy environment, reflected in initiatives such as Kudumbashree, the Kerala State Gender Policy 2023, and the Women Entrepreneurship Mission 2024 (Government of Kerala, 2025; Kerala Startup Mission, 2024), provides a distinctive setting for such an inquiry. The purpose of this study is to explore how government initiatives are associated with the relationship between employed women’s labour market engagement and their financial autonomy. This information is critical when attempting to ensure that women achieve substantive empowerment through work, along with gaining bargaining power and providing for the economic well-being of themselves and their families.

It is important to clarify the scope of this study at the outset. The sample consists exclusively of women who are already employed. Accordingly, the study does not examine the determinants of women’s entry into the labour force, and no comparison is made with women outside the labour market. The central construct, referred to in the model as LABPART, is therefore conceptualized and measured as *labour market engagement*—the intensity of employed women’s involvement in work, their confidence in their own employability, their perceived access to job opportunities, and their perception of professional growth through employment—rather than as labour force participation in the conventional labour-statistics sense. All hypotheses, results, and conclusions in this paper should be interpreted with reference to this construct and to the population of employed women in Kerala.

In light of this purpose, this study assesses how government initiatives mediate the association between employed women’s labour market engagement and their financial autonomy. By developing a comprehensive theoretical framework that encompasses cultural, economic, and institutional determinants, the study contributes to both theory and practice. The results of this research will be of assistance to policymakers, development agencies, and women’s organisations when developing future interventions that go beyond participation metrics, so that women have a true sense of control over finances, economic decisions, and their own lives, thus promoting sustainable and equitable development (UN Women, 2023).

The contribution of this study extends beyond confirming the established association between women’s employment and financial autonomy. Existing studies have largely examined women’s labour market participation, financial empowerment, and the outcomes of government programmes as separate areas of inquiry. Consequently, there is limited empirical evidence on the institutional mechanism through which labour market engagement is converted into women’s control over income, savings, and financial decisions. This study addresses that gap by modelling government initiatives as a mediator between labour market engagement and financial autonomy, and by estimating both the direct and indirect pathways within a unified structural framework. The study also contributes context-specific evidence from Kerala, where high levels of female education and social development coexist with relatively low labour force participation and continuing limitations on women’s economic agency. By focusing exclusively on employed women, the analysis further explains why engagement in work may be associated with differing levels of financial autonomy depending on the perceived accessibility and effectiveness of government support.

The rest of the paper is organised as follows. Section two describes the theoretical framework. Section three conducts a literature review and develops the hypotheses. Section four describes the methodology. Section five presents the results and discussion. Section six presents the conclusions, theoretical contributions, policy implications, and limitations of the study.

## 2 Theoretical framework

The idea of women’s engagement in the labour market has been debated in various theories such as Human Capital Theory, Empowerment Theory, and the Gender and Development (GAD) approach. Human Capital Theory, as proposed by Becker (1993), argues that investments in education, training, and health enhance the productivity and wages of people, which in turn stimulates economic growth in the country. From this, it can be derived that women’s engagement in the labour market is dependent on accumulated human capital, which determines their employability and earning capacity. Recent empirical findings have underscored that even if women accumulate substantial human capital, their engagement in the labour market and the gains from such engagement could be impeded by structural distortions such as informalization, skill underutilization, and wage discrimination (Mehrotra and Parida, 2022; Kocabıçak, 2025; Mishra and Sahoo, 2025; Chattopadhyay and Chowdhury, 2022).

Recent cross-country findings from sub-Saharan Africa and South Asia also validate the argument that an improvement in the level of education does not necessarily translate into a corresponding improvement in labour market outcomes or income autonomy (Thaddeus et al., 2022; Klasen and Pieters, 2022). Thus, education does not necessarily guarantee empowerment; instead, enabling institutional frameworks and balanced policies are needed to convert human capital into financial autonomy. The GAD Framework offers a complementary lens, highlighting that inequality rests in social and institutional structures rather than in deficits of individuals (Moser, 2012; Kabeer, 2021). This framework places women’s participation within the system of economic activities along contours of gendered power relations and norms prescribing role expectations, which control access to resources and opportunities. While Kerala boasts progressive social indicators and remnants of a more egalitarian matrilineal culture, patriarchal values prevail in labour segmentation and in decision-making authority, as can be seen from Centre for Public Policy Research (2024) and Ray Hajong and Kakarlapudi (2024). In this light, GAD theory explains why women, despite being educated, remain underrepresented in formal and high-skill sectors, since it shows that it is not market conditions alone that drive economic behaviour but cultural and institutional factors too. In addition, GAD also justifies the call for incorporating socio-cultural dimensions while analyzing labour market engagement and responsiveness to policy imperatives for promoting inclusion and empowerment (Jain and Chatterjee, 2024).

The Empowerment Theory provides the conceptual linkage between engagement and financial autonomy (Kabeer, 2001). Empowerment is defined as the process through which individuals gain the ability to make strategic life choices in contexts where this ability was previously denied. Economic engagement becomes meaningful only when it enhances women’s agency, control over income, and decision-making power within households and communities (Doss, 2023; UN Women, 2023). Financial autonomy, in that sense, ranges beyond income generation to embrace control over financial assets, savings, and investments. However, empirical research has shown that employment does not automatically translate into empowerment unless enabling policies—like equal-pay enforcement, skill development, childcare support, and entrepreneurship promotion—support this relationship (Economic Advisory Council to the Prime Minister, 2024; World Bank, 2023; Field et al., 2021). Initiatives in Kerala like Kudumbashree, the Gender Equality Policy 2023, and the Women Entrepreneurship Mission 2024 demonstrate policy mechanisms that may strengthen this mediating link between women’s work engagement and financial independence (Kudumbashree Mission, 2024).

In short, the three theories jointly motivate the constructs and pathways of the proposed model. Human Capital Theory underpins the economic determinants of engagement (wages, opportunities, incentives); the GAD Framework underpins the cultural determinants (social norms and family roles) and explains why institutional context matters; and Empowerment Theory links engagement to financial autonomy while identifying enabling policy as the mechanism through which work translates into control over resources. This mapping from theory to construct provides the justification for the hypothesized direct association between labour market engagement and financial autonomy (H1) and for the hypothesized mediating role of government initiatives (H2), developed in the next section.

## 3 Literature review and hypothesis development

### 3.1 Labour market engagement and financial autonomy

Women’s engagement in the labour market remains one of the central dimensions of economic inclusion and empowerment in the wider discourse on sustainable development and gender equality. Developmental economics regards women’s work as a pathway to productivity, which increases household income and creates spillovers for national output and human development (Klasen and Pieters, 2022; Jayachandran, 2021). However, persistent gender disparities in employment suggest that structural rigidities, occupational segregation, and limited access to quality employment restrict women’s economic agency (Chatterjee et al., 2023). Theoretically, investment in education and training should result in increased productivity and income according to the human capital approach, but the evidence shows that these benefits are not automatically channeled into autonomy or empowerment outcomes. Following Becker (1993) and Kocabıçak (2025), the gap between these two sets of outcomes indicates the presence of the institutional, social, and policy environments in which the individual exists and in which human capital is ultimately converted into material economic power (Chattopadhyay and Chowdhury, 2022; Kabeer and Natali, 2023). Recent studies from the Journal of Business and Socio-economic Development also indicate that structural inequalities, such as ownership concentration and governance asymmetry, can offer a moderating effect on the relationship between participation and empowerment outcomes. At least part of the effectiveness of translating individual engagement into socio-economic returns seems to depend on institutional legitimacy and stakeholder inclusiveness (Al Amosh and Khatib, 2022).

Empowerment Theory positions financial autonomy as involving more than the actual receipt of income: decision-making authority, savings, and financial security through the control of investment. Women who are more actively engaged in the labour market accumulate financial assets and thus have more bargaining power in the household over intra-household distributional decisions and economic strategies (Field et al., 2021; Duflo, 2012).

The magnitude of this association varies according to the quality and security of work opportunities; insecure or informal employment offers little or no agency gain (Kabeer, 2021; Heath and Jayachandran, 2018). Drawing these strands together, Empowerment Theory implies that the intensity of employed women’s engagement with the labour market—their involvement, employability confidence, and perceived professional growth—should be positively associated with the degree of control they exercise over income, savings, and financial decisions. This theoretical expectation, supported by the empirical literature reviewed above, leads to the first hypothesis:

*H1*: Women’s labour market engagement has a positive and significant association with financial autonomy.

### 3.2 Government initiatives, labour market engagement and financial autonomy

While labour market engagement initiates the empowerment process, it is often government interventions that determine whether engagement translates into sustainable autonomy. Policy frameworks, in congruence with stakeholder and legitimacy theories applied in JBSED research, serve as mediating mechanisms that align social objectives with economic participation by institutionalizing gender equity through legislation, incentives, and public programs (Agarwal, 2023; Deininger et al., 2022). Government-led initiatives such as gender-responsive budgeting, microfinance, entrepreneurship promotion, skill development missions, and social-security reforms create an enabling environment within which women’s economic activities can evolve into financial empowerment (Garikipati and Kambhampati, 2023; Suri and Jack, 2023). Empirical studies show that public interventions mitigate market failures bound to affect women disproportionately, such as limited access to credit facilities, wage discrimination, and unequal social protection (Buvinic and O’Donnell, 2023; Bandiera et al., 2020). The Journal of Business and Socio-economic Development also shows that institutional independence with transparent governance mechanisms strengthens the accountability of such interventions and their potential for transformative changes. Legitimacy and empowerment perspectives would, therefore, integrate to indicate that government policies are not merely complementary but rather mediating variables between women’s engagement and their financial autonomy (Kabeer and Santos, 2024).

Despite growing evidence that labour market engagement can improve women’s economic agency, and that public programmes can enhance access to skills, credit, networks, and social protection, these two streams of literature remain insufficiently integrated. Previous studies have predominantly estimated the direct association between employment and empowerment, or evaluated the independent outcomes of particular government programmes. They provide limited evidence on whether government initiatives constitute an intervening institutional pathway through which employed women’s labour market engagement is associated with financial autonomy. This gap is particularly relevant in Kerala, where extensive gender-oriented programmes operate alongside persistent disparities between women’s educational attainment, employment engagement, and control over financial resources. The present study addresses this gap by testing government initiatives as a mediator, rather than treating them merely as an external policy condition or an additional explanatory variable.

Addressing this gap, and consistent with the GAD and Empowerment perspectives, the study proposes that employed women who are more strongly engaged in the labour market may be better positioned to become aware of, access, and benefit from government-sponsored programmes (engagement → initiatives). The skills, credit, networks, employment support, and confidence generated through these initiatives may subsequently strengthen women’s control over income, savings, and financial decisions (initiatives → autonomy). Government initiatives are therefore conceptualised as an intervening institutional mechanism linking labour market engagement with financial autonomy. This reasoning leads to the second hypothesis:

*H2*: Government initiatives significantly mediate the association between women’s labour market engagement and financial autonomy.

## 4 Research methodology

### 4.1 Population, sampling frame and sample

The reference population of the present study consisted of all employed women in Kerala as estimated by the Periodic Labour Force Survey (PLFS) 2023–24, conducted by the Ministry of Statistics and Programme Implementation (2024). According to the PLFS estimates, the total female workforce in Kerala is around 7.6 million, employed across formal, informal, and self-employed sectors. Because no single register of all employed women exists, the sampling frame comprised women workers in the five selected districts who were accessible through Self-Help Groups (SHGs), Kudumbashree neighbourhood groups, and the researchers’ professional and community networks. These community-based platforms provided access to women engaged in diverse economic activities—including self-employment, entrepreneurship, agriculture, services, and informal sector occupations—and enabled the identification of eligible respondents from varied socio-economic backgrounds.

A multi-stage stratified random sampling procedure was adopted. In the first stage, five districts—Kozhikode, Wayanad, Ernakulam, Alappuzha, and Thiruvananthapuram—were purposively selected to represent the northern, central, and southern socio-economic regions of Kerala. In the second stage, respondents within each district were stratified according to the nature of their jobs into three categories—skilled, semi-skilled, and unskilled—based on the National Classification of Occupations (NCO) 2015, and respondents were selected at random within each stratum in proportion to the stratum’s estimated share of the district workforce. The target sample of 1,500 respondents was allocated across the five districts in proportion to each district’s estimated female workforce, based on Census of India (2011) and PLFS (Ministry of Statistics and Programme Implementation, 2024) figures; the planned district-wise allocation is reported in Appendix A (Table A1).

Regarding sample size, the final analytical sample of 1,357 respondents drawn from a population of approximately 7.6 million yields a margin of error of approximately ±2.7 percentage points at the 95 percent confidence level (assuming maximum variability, *p* = 0.5), which substantially exceeds the precision of the conventional Cochran minimum of 384 respondents. The sample of 1,357 was therefore judged adequate for Structural Equation Modelling, which typically requires large samples for stable estimation (Byrne, 2016; Hair et al., 2021).

Primary data were collected through a structured questionnaire administered through these community-based platforms over a three-month period between February and April 2025. Respondents were informed about the objectives of the study, participation was entirely voluntary, and confidentiality and anonymity were assured throughout the data collection process. A total of 1,650 questionnaires were distributed, of which 1,540 were returned. After excluding 40 incomplete questionnaires, 1,500 valid responses were obtained, yielding an effective response rate of 90.9 percent. Data screening prior to estimation led to the exclusion of a further 143 responses with excessive missing values or inconsistent answer patterns, so that the final analytical sample comprised 1,357 usable responses (82.2 percent of the questionnaires distributed).

Of the 1,357 respondents, 550 (40.5%) belonged to the skilled category, including teachers, nurses, office administrators, accountants, and technical assistants. The semi-skilled group comprised 479 respondents (35.3%), primarily engaged as sales staff, tailoring assistants, receptionists, clerks, and field workers. A total of 328 respondents (24.2%) were unskilled, including domestic workers, cleaning staff, helpers, and daily-wage labourers. In terms of employment status, 234 respondents (17.2%) were employed in the government sector, 1,037 (76.4%) in the private sector, and 86 (6.3%) were self-employed. Table 1 presents the composition of the sample by occupational category, skill level, and employment status.

**Table 1:** Composition of the sample by worker category.

Skill category	Representative occupations	Freq.	%
Skilled	Teachers, nurses, office administrators, accountants, technical assistants	550	40.5
Semi-skilled	Sales staff, tailoring assistants, receptionists, clerks, field workers	479	35.3
Unskilled	Domestic workers, cleaning staff, helpers, daily-wage labourers	328	24.2
Total		1,357	100.0
Employment status			
Government sector	Regular salaried public employment	234	17.2
Private sector	Salaried and wage employment in private establishments	1,037	76.4
Self-employed	Own-account workers and micro entrepreneurs	86	6.3
Total		1,357	100.0

Source: Primary data. Skill categories follow the National Classification of Occupations (NCO) 2015. Government and private sector categories include both permanent and casual arrangements; self-employment and much of the unskilled category correspond to informal work.

The deliberate inclusion of semi-skilled and unskilled workers requires explanation. Informal and low-skilled employment accounts for the overwhelming majority of female work in India, and restricting the sample to skilled or formally employed women would have produced a systematically unrepresentative account of women’s economic engagement in Kerala. Excluding these categories would also have removed precisely those women for whom government initiatives are most consequential, since programmes such as Kudumbashree are designed principally for women in informal and low-income occupations. Including the full skill spectrum therefore permits the mediating role of government initiatives to be examined across the range of employment conditions in which women actually work, rather than only among those already in secure formal employment. The interpretive limits that follow from including informal workers, whose financial autonomy is constrained by irregular earnings and the absence of social security, are acknowledged in Section 6.3.

### 4.2 Instrument development and measures

All constructs were measured with multi-item scales on a five-point Likert format (1 = strongly disagree to 5 = strongly agree). Items were adapted from established scales and frameworks in the prior literature and refined to the Kerala context: cultural determinants from the World Values Survey and ILO gender-equality studies; economic determinants from ILO and World Bank gender statistics frameworks; labour market engagement from ILO labour force survey concepts; financial autonomy from Kabeer (1999), Malhotra et al. (2002), and the World Bank women’s empowerment framework; and government initiatives from Kudumbashree Mission reports, Ministry of Women and Child Development documents, and the NITI Aayog Women Entrepreneurship Platform (a construct-wise summary of measurement sources is provided in Appendix A). The questionnaire was originally prepared in English, translated into Malayalam, and back-translated by independent bilingual experts to ensure semantic equivalence. Prior to the main survey, the instrument was pilot-tested on 150 women workers (10 percent of the intended sample), who were excluded from the final sample, to assess the clarity, reliability, and feasibility of the questionnaire; based on the pilot findings, minor revisions were made to the wording and sequencing of selected items before the final survey was undertaken. The full list of items for each construct is provided in Appendix A.

Labour Market Engagement (retaining the model code LABPART for consistency with the estimation output) captures employed women’s active involvement in the labour market, confidence in employability, perceived access to job opportunities, and perception of professional growth through employment. It is emphasized that this construct does not measure labour force participation in the conventional labour-statistics sense (i.e., the decision to enter or remain in the labour force), since all respondents are already employed.

### 4.3 Data analysis

The SEM technique was applied in the current study to analyze the interrelationships among the latent constructs Cultural Determinants, Economic Determinants, Labour Market Engagement, Government Initiatives, and Financial Autonomy using AMOS 28.0. The method allows both the measurement model (confirmatory factor analysis) and the structural model (path analysis) to be tested simultaneously for a comprehensive validation of the theoretical framework, as discussed by Hair et al. (2021) and Byrne (2016). Before estimating the SEM, reliability and validity analyses were performed for all constructs. All latent variables had Cronbach’s alpha values greater than 0.80 (ranging from 0.802 to 0.953), which established internal consistency. The Kaiser–Meyer–Olkin test value of 0.87 and Bartlett’s Test of Sphericity (*p* < 0.001) confirmed that the sampling was adequate and suitable for factor analysis. The Average Variance Extracted (AVE) values fell between 0.592 and 0.781, thus meeting convergent validity, while Composite Reliability exceeded the threshold of 0.70 in all the constructs (0.851 to 0.962) (Fornell and Larcker, 1981). Construct-wise reliability and validity statistics are reported in Table 2.

**Table 2:** Construct reliability and validity.

Construct	Items	Cronbach’s *α*	CR	AVE
Cultural determinants	8	0.802	0.851	0.592
Economic determinants	9	0.859	0.892	0.624
Labour market engagement	5	0.822	0.867	0.611
Government initiatives	5	0.953	0.962	0.781
Financial autonomy	9	0.899	0.917	0.690

Source: Primary data. CR, Composite Reliability; AVE, Average Variance Extracted. Discriminant validity was assessed using the Fornell–Larcker criterion.

Because all data were self-reported and collected from a single source at a single point in time, the potential for common method bias was assessed using Harman’s single-factor test, which suggested that common method bias is unlikely to be a serious concern. Nevertheless, self-report bias cannot be fully ruled out, and this is acknowledged in the limitations.

The mediation hypothesis (H2) was tested formally by estimating the direct, indirect, and total effects of Labour Market Engagement on Financial Autonomy, with the indirect effect assessed using bias-corrected bootstrapping with 5,000 resamples and 95 percent confidence intervals in AMOS. Mediation is inferred when the bootstrapped confidence interval for the indirect effect excludes zero.

### 4.4 Research model

Based on the theoretical framework, the study’s conceptual and structural model is presented in Figure 1, depicting the hypothesized pathways among the constructs.

**Figure 1:**
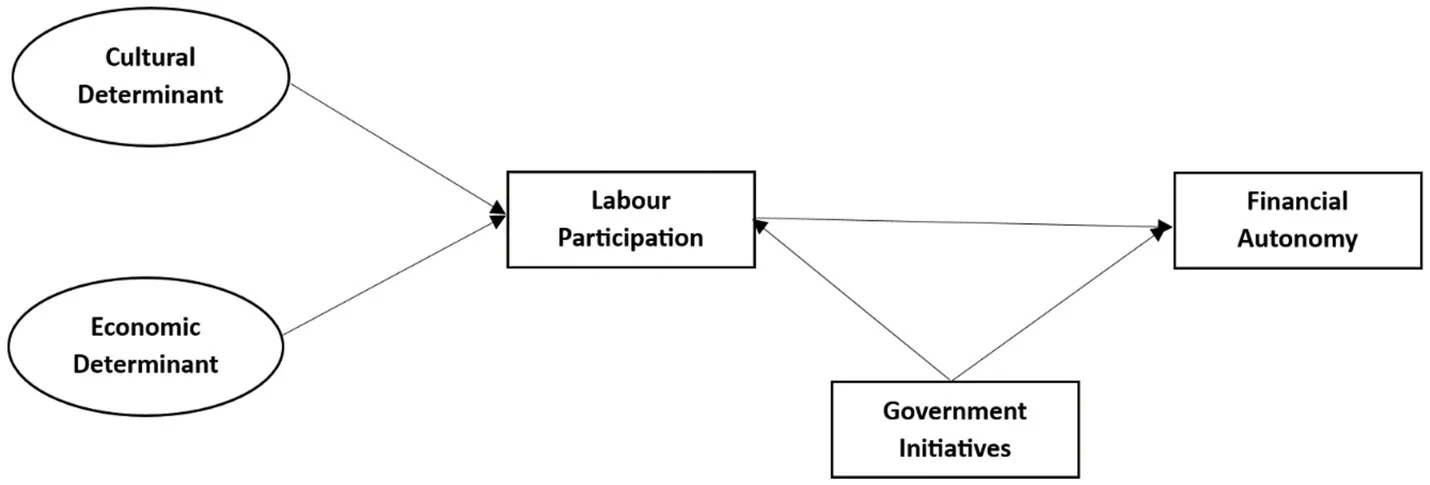
Conceptual model.

The model integrates both cultural and economic determinants as antecedent variables associated with Labour Market Engagement, which in turn is associated with Financial Autonomy. The Government Initiatives construct acts as a mediating variable to capture the indirect association between labour market engagement and financial outcomes. Two regression equations have been developed to represent the direct and mediated pathways within the SEM framework (see Table 3).

**Table 3:** The study variable description.

Variable	Code	Data coverage
Cultural determinants	CULTDET	Includes *social norms* (attitudes toward women’s leadership, gender equality, shared domestic roles) and *family role* (family encouragement, household responsibility sharing, family support for women’s careers).
Economic determinants	ECONDET	Comprises *economic factors* (financial incentives, cost of living, employment benefits), *employment opportunities* (availability and suitability of jobs for women), and *wage rates* (fairness, competitiveness, equality).
Labour market engagement	LABPART	Reflects employed women’s active engagement in the labour market, confidence in employability, access to job opportunities, and perception of professional growth through employment.
Financial autonomy	FINAUTO	Represents women’s control over personal and household income, independent financial decision making, savings, and financial confidence.
Effectiveness of government initiatives	GOVINIT	Covers perceptions of the effectiveness of government programs in enhancing skills, confidence, social engagement, and financial stability.

#### 4.4.1 Regression model

The following regression model is developed to examine the mediating role of Government Initiatives in the association between labour market engagement and financial autonomy:

$$\begin{array}{l} \text{FINAUT}O_{i}=\alpha +\beta_{1}\text{CULTDE}T_{i}+\beta_{2}\text{ECONDE}T_{i}\\ +\beta_{3}\text{LABPAR}T_{i}++\beta_{4}\text{GOVINI}T_{i}+\varepsilon_{i} \end{array}$$

## 5 Results and discussion

### 5.1 Demographic profile of the sample

The demographic profile of the respondents gives an idea about the composition of women workers in the present study. Almost half of the respondents (47.8%) fall into the 18–30-year group, indicating that younger women form a large share of the employed sample. This reflects changing socio-economic aspirations and better educational attainment among women, as already reported in the literature by Ray Hajong and Kakarlapudi (2024) and International Labour Organization (2024). This concentration is consistent with the age structure of Kerala’s female workforce rather than an artefact of sampling: younger cohorts have entered paid employment in greater numbers following near-universal female schooling, while participation among older cohorts remains depressed by the withdrawal patterns associated with marriage and caregiving that have historically characterised the state. The predominance of younger respondents therefore reflects who is actually employed in Kerala, and it is precisely the cohort for whom questions of financial autonomy and access to government programmes are most immediately consequential. Nevertheless, the age profile of the sample means that the findings speak most directly to women in the early and middle stages of their working lives, and this is noted among the limitations in Section 6.3. A considerable number of the respondents were married, accounting for 59.9 percent, suggesting that economic necessity and dual-income households have become significant reasons for women’s continued presence in the workforce. However, this percentage also represents the double burden many married women face in balancing work obligations with domestic responsibilities—a pattern observed across India, as stated by Jayachandran (2021) and Ministry of Statistics and Programme Implementation (2024). Occupationally, a majority of the respondents (76.4%) are employed in the private sector, reflecting the dominance of service-oriented employment in Kerala’s labour market. The government sector contributes 17.2%, while only 6.3% are self-employed. In terms of nature of employment, an appreciable share of the respondents (40.5%) are skilled, reflecting the gradual enhancement of skills and increased female presence in semi-professional categories. However, quite a significant section remains in semi-skilled and unskilled work, reflecting continued structural barriers in career advancement (World Bank, 2023). Besides, the predominance of nuclear families (76.9%) reflects the changes in social structures in Kerala, which may be associated with more independent decision-making among employed women (Thomas, 2022) (see Table 4).

**Table 4:** Demographic profile of women workers.

Variables		Frequency	Percent	Cumulative percent
Age (years)	18–30	648	47.80	47.80
	31–40	481	35.40	83.20
	41–50	174	12.80	96.00
	51–60	54	4.00	100.00
	Total	1,357	100.00	
Marital status	Unmarried	448	33.00	33.00
	Married	813	59.90	92.90
	Separated	57	4.20	97.10
	Widowed	39	2.90	100.00
	Total	1,357	100.00	
Occupation	Self employed	86	6.30	6.30
	Private	1,037	76.40	82.80
	Government	234	17.20	100.00
	Total	1,357	100.00	
Job nature	Skilled	550	40.50	40.50
	Semi skilled	479	35.30	75.80
	Unskilled	328	24.20	100.00
	Total	1,357	100.00	
Type of family	Joint family	314	23.10	23.10
	Nuclear family	1,043	76.90	100.00
	Total	1,357	100.00	
Type of residence	Own house	1,148	84.60	84.60
	Rented	197	14.50	99.10
	Other	12	0.90	100.00
	Total	1,357	100.00	

Source: Primary data.

### 5.2 Descriptive statistics

Table 5 shows the summary of the variables’ descriptive statistics. The analysis shows that the mean values range between 2.86 and 3.27, indicating that most respondents expressed moderate to moderately positive perceptions across the constructs. The findings reveal that Economic Determinants recorded a relatively high mean score (Mean = 3.23, SD = 0.73), suggesting that respondents moderately agreed that factors such as wage rates, job availability, and financial incentives are important considerations in their labour market engagement. This implies that economic motivators and employment opportunities remain key correlates of women’s engagement in paid work, consistent with earlier findings in labour economics literature (International Labour Organization, 2024; Mehrotra and Parida, 2022). The mean score for Labour Market Engagement (Mean = 3.27, SD = 1.09) shows that the women in the sample display a generally positive orientation toward workforce involvement. Respondents indicated confidence in their employability and motivation to pursue professional growth, although variation across job categories (skilled, semi-skilled, and unskilled) suggests differences in the quality and stability of employment. Cultural Determinants reported a mean of 3.08 (SD = 1.17), implying that cultural and familial influences continue to shape women’s work-related decisions. While social support for women’s careers has improved, traditional gender norms and family expectations still moderate economic choices (Jayachandran, 2021; Centre for Public Policy Research, 2024). The average for Financial Autonomy (Mean = 3.06, SD = 1.22) indicates that most women experience a moderate level of control over their income and financial decisions. This finding aligns with prior research emphasizing that employment alone is not automatically accompanied by empowerment unless supported by policy and equal pay structures (Kabeer, 2021; Doss, 2023). Additionally, the Effectiveness of Government Initiatives yielded the lowest mean (Mean = 2.86, SD = 0.93), suggesting that although several women-focused programmes such as Kudumbashree and the Women Entrepreneurship Mission are operational, their perceived reach remains limited. The relatively lower mean underscores the need for greater policy outreach, improved training, and better linkage between government schemes and women’s financial independence (Economic Advisory Council to the Prime Minister, 2024; UN Women, 2023) (see Table 6).

**Table 5:** Descriptive results.

Variables	Obs	Minimum	Maximum	Mean	S.D.
CULTDET	1,357	1	5	3.08	1.17
ECONDET	1,357	1	5	3.23	0.73
LABPART	1,357	1	5	3.27	1.09
FINAUTO	1,357	1	5	3.06	1.22
GOVINIT	1,357	1	5	2.86	0.93

Source: Primary data.

**Table 6:** Model fit for SEM.

Indices	Value	Suggested value
Chi-square value	50.101	–
*df*	18	–
*p*-value	< 0.001	> 0.05 (Hair et al., 1998)
Chi-square value/*df*	2.783	< 5.00 (Hair et al., 1998)
GFI	0.978	> 0.90 (Hu and Bentler, 1999)
NFI	0.928	> 0.90 (Hu and Bentler, 1999)
TLI	0.936	> 0.90 (Hu and Bentler, 1999)
CFI	0.949	> 0.90 (Hooper et al., 2008)
RMR	0.040	< 0.08 (Hair et al., 2006)
RMSEA	0.057	< 0.08 (Hair et al., 2006)

Source: Primary data.

### 5.3 Model fit and structural model results

The SEM model was evaluated using the Chi-square statistic, Goodness of Fit Index (GFI), Normed Fit Index (NFI), Tucker–Lewis Index (TLI), Comparative Fit Index (CFI), Root Mean Square Residual (RMR) and Root Mean Square Error of Approximation (RMSEA). Although the chi-square statistic was significant (*χ*^2^ = 50.101, df = 18, *p* < 0.001), the chi-square test is well known to be sensitive to large sample sizes such as the present one (*n* = 1,357), and reliance is therefore placed on the normed chi-square and the incremental and absolute fit indices (Hooper et al., 2008). The normed chi-square (*χ*^2^/df = 2.783) and all remaining indices fall within their respective recommended thresholds, indicating that the model demonstrated an acceptable fit to the data (Table 5).

Figure 2 presents the estimated structural model using AMOS 28.0, which shows the hypothesized associations between Cultural Determinants, Economic Determinants, Labour Market Engagement, Government Initiatives, and Financial Autonomy. Based on the fit indices reported in Table 5, the hypothesized model fits the data acceptably and can be considered to describe the observed relationships among constructs satisfactorily.

**Figure 2:**
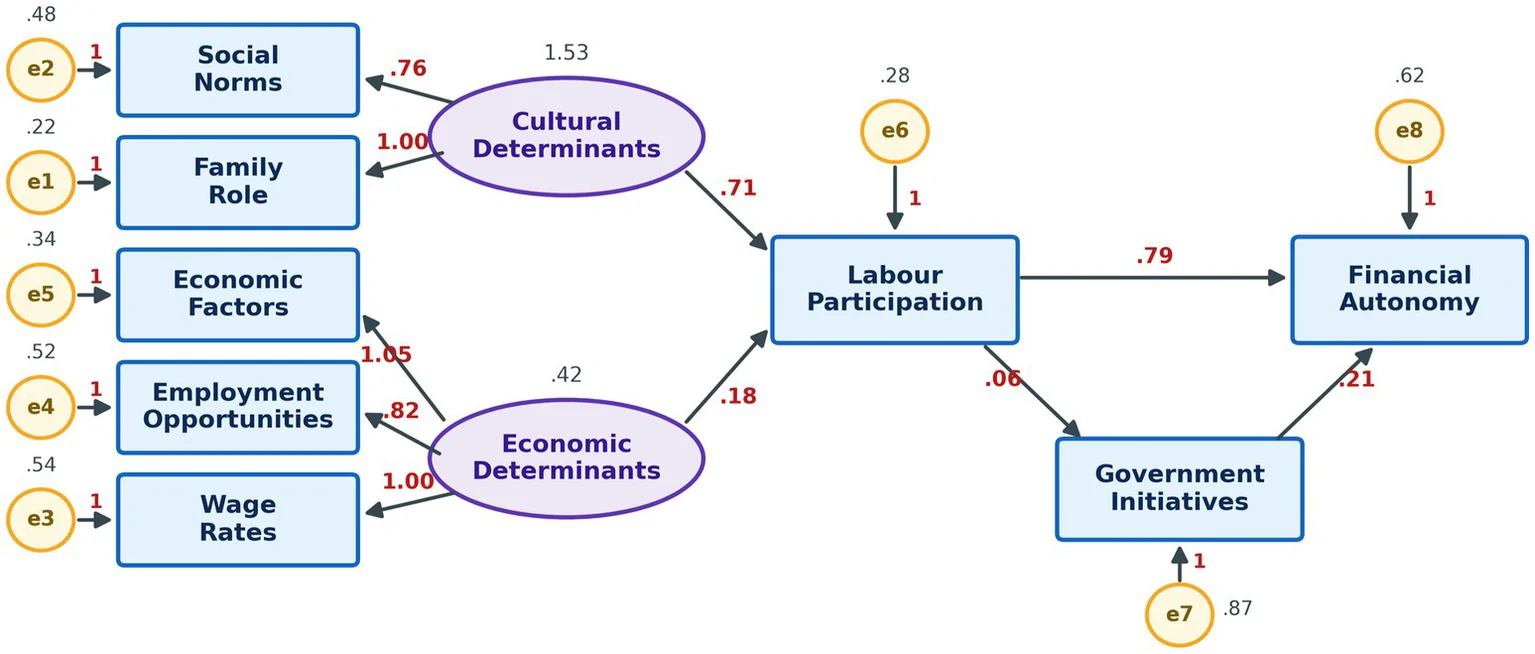
Structural model results.

The path coefficients resulting from the model indicate that Cultural Determinants have a strong positive and significant association with Labour Market Engagement (*β* = 0.85, *p* < 0.001). Such a result indicates that socio-cultural factors, like family role expectations and social norms prevalent in society, are strongly related to women’s engagement in paid employment. A facilitating culture—for instance, where the family encourages female education and work—may be positively associated with women’s labour market engagement. This finding is in agreement with the results of Jayachandran (2021) and Centre for Public Policy Research (2024), who noted that changing gender attitudes in Kerala have started to be positively associated with women’s work participation, notwithstanding lingering traditional pressures.

On the other hand, Economic Determinants turned out to have a weak but positive association with Labour Market Engagement (*β* = 0.11, *p* < 0.05). This suggests that although wage rates, employment opportunities, and economic incentives are relevant to women’s engagement in the labour market, their relative association is less salient than that of cultural factors. This modest strength indicates that favorable economic conditions may not correspond with higher engagement unless reinforced by cultural acceptance and supportive work environments. Similar patterns have been reported in other developing economies where socio-cultural barriers outweigh economic motivators (Mehrotra and Parida, 2022; International Labour Organization, 2024).

The analysis further reveals that Labour Market Engagement has a strong positive and significant association with Financial Autonomy (*β* = 0.74, *p* < 0.001), thus supporting Hypothesis 1 (H1). This indicates that women who are more engaged in the labour market report higher financial independence, decision-making authority, and resource control. Employment is associated with women’s access to income, savings, and credit, and correspondingly with improved bargaining power within the household. This is consistent with several empirical studies conducted earlier, which have emphasized the link between female workforce engagement and women’s economic empowerment (Kabeer, 2021; Doss, 2023).

### 5.4 Mediation analysis

Hypothesis 2 proposed that Government Initiatives mediate the association between Labour Market Engagement and Financial Autonomy. The component paths were both significant: the path from Labour Market Engagement to Government Initiatives was *β* = 0.06 (*p* < 0.05), and the path from Government Initiatives to Financial Autonomy was *β* = 0.17 (*p* < 0.05). However, the significance of the component paths alone does not establish mediation. Accordingly, the indirect effect was tested formally using bias-corrected bootstrapping with 5,000 resamples in AMOS. Table 7 reports the standardized direct, indirect, and total effects with their 95 percent bootstrapped confidence intervals.

**Table 7:** Direct, indirect and total effects (standardized), with 95% bias-corrected bootstrapped confidence intervals (5,000 resamples).

Effect (LABPART → FINAUTO)	Estimate	95% CI [Lower, Upper]	*p*
Direct effect	0.740	[0.621, 0.843]	< 0.001
Indirect effect (via GOVINIT; 0.06 × 0.17)	0.010	[0.003, 0.024]	0.021
Total effect	0.750	[0.635, 0.852]	< 0.001

Source: Primary Data. Mediation is inferred only where the bootstrapped confidence interval for the indirect effect excludes zero.

The bootstrapped indirect effect of Labour Market Engagement on Financial Autonomy through Government Initiatives was positive and statistically significant [standardized indirect effect = 0.010, 95% CI (0.003, 0.024), *p* = 0.021]. Since the confidence interval excludes zero while the direct effect remains large and significant [0.740, 95% CI (0.621, 0.843)], the results support Hypothesis 2 and indicate *partial* mediation, albeit of modest magnitude: the indirect pathway accounts for approximately 1.3 percent of the total effect [0.750, 95% CI (0.635, 0.852)]. These results suggest that policy-driven mechanisms such as microfinance schemes, entrepreneurship development programs, and training under Kudumbashree and the Women Entrepreneurship Mission (Kudumbashree Mission, 2024) are associated with the translation of labour market engagement into financial empowerment, although the bulk of the association between engagement and autonomy is direct. The modest size of the indirect association is itself informative for policy: it suggests considerable unrealized scope for government initiatives to strengthen the link between women’s work and their financial autonomy, consistent with the comparatively low perceived effectiveness of these programs reported in Table 5. Because the study is cross-sectional and based on self-reported data, these findings indicate associations consistent with the hypothesized mediation mechanism; they do not establish causal program effects.

The *R*^2^ values indicate that the model explains 74% of the variance in Financial Autonomy, whereas Cultural and Economic Determinants jointly explain 68% of the variance in Labour Market Engagement. The high explanatory power indicates that these variables capture important socio-economic and policy-related correlates of women’s financial empowerment in Kerala.

## 6 Conclusion

This study explored the associations among cultural norms, economic conditions, labour market engagement, government initiatives, and the financial autonomy of employed women in Kerala, India, with particular attention to the mediating role of government interventions established for women’s empowerment. By integrating cultural, economic, and institutional elements into a Structural Equation Modelling framework, the study empirically examined how socio-cultural determinants, economic resources, and policy interventions are associated with women’s economic empowerment among those already in the workforce.

The study concludes that, within this sample of employed women, cultural factors carry greater weight than economic factors in their association with labour market engagement. One reason for this is the persistent influence of family, gender, and social expectations on the lives of working women. While wages, job opportunities, and similar economic considerations matter to women, they exist within the overall context of culturally approved and accepted ways of working. Thus, it appears necessary for structural changes to take place simultaneously in women’s employment practices and in women’s roles in the family. The study also finds a direct, significant association between women’s labour market engagement and their financial autonomy, suggesting that the more intensively women engage with the workforce, the greater their reported control over income, assets, and decision-making. However, the direct association is only part of the total association between engagement and financial independence, with government initiatives accounting for a modest indirect component.

The explanatory power of the model for Financial Autonomy (*R*^2^ = 0.74) supports the argument that sustainable empowerment through employment alone is unlikely without adequate institutional and policy design support. The findings indicate that government program efficacy should be enhanced through improved outreach, gender budgeting, and constant monitoring to ensure benefits are provided to women at all levels of employment, including semi-skilled and unskilled workers.

It bears repeating that, because the sample comprises employed women only, these conclusions pertain to the intensity and quality of engagement among women who are already in work, and to the financial autonomy associated with that engagement. The study does not speak to the determinants of women’s entry into the labour force.

### 6.1 Theoretical contributions

The research contributes to gendered labour economics in three specific ways. First, it integrates Human Capital Theory, the Gender and Development Framework, and Empowerment Theory into a single estimable model, showing that each theory maps onto a distinct construct—economic determinants, cultural determinants, and the engagement–autonomy pathway, respectively, —and that their joint specification explains substantially more variance in financial autonomy than any single-theory account would suggest. Second, by distinguishing *labour market engagement* among employed women from labour force participation in the conventional sense, the study offers a construct suited to intensive-margin research on women’s work in contexts, such as Kerala, where the analytically interesting variation lies among women already in employment. Third, the finding that cultural determinants are far more strongly associated with engagement than economic determinants (*β* = 0.85 versus *β* = 0.11) provides quantitative support, in a high-human-development Indian state, for the GAD proposition that institutional and normative structures, rather than individual economic incentives alone, condition women’s economic behaviour.

### 6.2 Policy implications

The findings carry specific implications for policy design in Kerala. The comparatively low perceived effectiveness of government initiatives (mean = 2.86, the lowest of all constructs) alongside their significant but modest mediating association suggests that the existing architecture—Kudumbashree, the Kerala State Gender Policy 2023, and the Women Entrepreneurship Mission—is reaching working women only partially. Three directions follow. First, outreach and last-mile delivery should be strengthened for semi-skilled and unskilled workers, who form nearly 60 percent of the sample and are least likely to be connected to formal schemes. Second, given the dominance of cultural determinants, programs should incorporate family- and community-facing components—engaging households in training and entrepreneurship schemes—rather than targeting women in isolation. Third, scheme design should link employment support explicitly to financial-control outcomes (individual bank accounts, savings products, and financial-literacy modules embedded in skill programs), since the results indicate that engagement is most strongly associated with autonomy when institutional mechanisms support the conversion of income into control over resources.

### 6.3 Limitations and future research

Several limitations should be acknowledged when interpreting these findings. First, the research design is cross-sectional; therefore, the estimated paths represent empirical associations and no causal inferences can be drawn. Longitudinal or panel designs would be required to examine whether changes in engagement precede changes in financial autonomy. Second, the sample comprises employed women only, so the findings cannot be generalized to women outside the labour force, and the study is silent on labour-force entry. Third, all constructs were measured through self-reported perceptions, which raises the possibility of common method and social-desirability bias; although Harman’s single-factor test did not indicate a serious problem, future research could triangulate perceptions with administrative or behavioural data. Fourth, the sampling frame relied on Self-Help Groups, Kudumbashree neighbourhood groups, and community networks; while this enabled access to a large and diverse pool of women workers, it may under-represent employed women who are not connected to such networks, and this potential frame bias should be borne in mind. Fifth, the sample spans the full skill spectrum, including women in informal and daily-wage occupations. While their inclusion is essential to a representative account of female employment in Kerala, financial autonomy in these categories is constrained by irregular earnings, the absence of written contracts, and limited access to social security, so that the autonomy reported by informal workers should be understood as relative control over modest and unstable income rather than as financial security in the fuller sense. Sixth, respondents were concentrated in the 18–30 and 31–40 age groups; although this mirrors the age composition of Kerala’s female workforce, the findings apply most directly to women in the early and middle stages of their working lives, and generalisation to older cohorts, whose employment histories and household positions differ, should be made with caution. Seventh, the sample, while large and stratified, was drawn from five districts of Kerala, a state with atypically high female literacy and social development indicators; generalization beyond Kerala, and to India as a whole, should therefore be made with caution. Eighth, the mediating role of government initiatives was measured through perceived program effectiveness rather than verified program participation; future studies could use scheme-level administrative data to examine specific programs. Finally, future research could extend the model with moderating variables such as job formality, sector, and household structure, and could replicate the framework in states with contrasting socio-cultural profiles.

## Data Availability

The raw data supporting the conclusions of this article will be made available by the authors, without undue reservation.

## References

[ref1] Agarwal B. (2023). Gender equality, governance, and economic empowerment. World Dev. 165:106189.

[ref2] Al Amosh H. Khatib S. F. A. (2022). Ownership structure and environmental, social and governance performance disclosure: the moderating role of the board independence. J. Bus. Soc. Econ. Dev. 2, 49–66. doi: 10.1108/JBSED-07-2021-0094

[ref3] Bandiera O. Buehren N. Burgess R. Goldstein M. Gulesci S. Rasul I. . (2020). Women’s empowerment in action: evidence from a randomized control trial in Africa. Am. Econ. J. Appl. Econ. 12, 210–259. doi: 10.1257/app.20170416

[ref4] Becker G. S. (1993). Human Capital: a Theoretical and Empirical Analysis, with Special Reference to Education. 3rd Edn. Chicago: University of Chicago Press.

[ref5] Buvinic M. O’Donnell M. (2023). Gender matters in economic development: policy interventions and outcomes. World Dev. 168:106287.

[ref6] Byrne B. M. (2016). Structural Equation Modeling with AMOS: Basic Concepts, Applications, and Programming. 3rd Edn. New York: Routledge.

[ref7] Census of India (2011). Primary Census Abstract. Office of the Registrar General & Census Commissioner. New Delhi: Government of India.

[ref8] Centre for Public Policy Research (2024) Kerala’s patriarchal dominance and low female workforce participation. Available online at: https://www.cppr.in/articles/keralas-patriarchal-dominance

[ref9] Chatterjee U. Murgai R. Rama M. (2023). Job opportunities along the rural-urban gradation and female labour force participation in India. World Dev. 163:106156.

[ref10] Chattopadhyay S. Chowdhury S. (2022). Female labour force participation in India: trends, drivers and barriers. Indian J. Labour Econ. 65, 1–23. doi: 10.1007/s41027-022-00362-0

[ref11] Deininger K. Jin S. Nagarajan H. (2022). Women’s land rights and labour market outcomes. J. Dev. Econ. 157:102829.

[ref12] Doss C. (2023). Women’s control over income and household decision-making: evidence from developing countries. World Dev. 166:106199.

[ref13] Duflo E. (2012). Women empowerment and economic development. J. Econ. Lit. 50, 1051–1079. doi: 10.1257/jel.50.4.1051

[ref14] Economic Advisory Council to the Prime Minister (2024). Female Labour Force Participation Rate—India (Working Paper). New Delhi: Government of India.

[ref15] Elborgh-Woytek K. Newiak M. Kochhar K. Fabrizio S. Kpodar K. Wingender P. . (2013). Women, Work, and the Economy: Macroeconomic Gains from Gender Equity. Washington, DC: International Monetary Fund.

[ref16] Field E. Pande R. Rigol N. Schaner S. Troyer Moore C. (2021). On her own account: how strengthening women’s financial control impacts labor supply and gender norms. Am. Econ. Rev. 111, 2342–2375. doi: 10.1257/aer.20200705 PMC10191162 37201159

[ref17] Fornell C. Larcker D. F. (1981). Evaluating structural equation models with unobservable variables and measurement error. J. Mark. Res. 18, 39–50. doi: 10.1177/002224378101800104

[ref18] Garikipati S. Kambhampati U. (2023). Microfinance and women’s empowerment: evidence and insights. World Dev. 161:106068.

[ref19] Goldin C. (1995). “The U-shaped female labour force function in economic development,” in Investment in Women’s Human Capital and Economic Development, ed. Schultz T. P. (Chicago: University of Chicago Press), 61–90.

[ref20] Government of Kerala (2025). Gender Budget 2025–26. Upper Saddle River, NJ: Thiruvananthapuram: Government of Kerala.

[ref21] Hair J. F. Anderson R. E. Tatham R. L. Black W. C. (1998). Multivariate Data Analysis. 5th Edn. Upper Saddle River, NJ: Prentice Hall.

[ref22] Hair J. F. Black W. C. Babin B. J. Anderson R. E. Tatham R. L. (2006). Multivariate Data Analysis. 6th Edn. Upper Saddle River, NJ: Pearson Prentice Hall.

[ref23] Hair J. F. Hult G. T. M. Ringle C. M. Sarstedt M. (2021). A Primer on Partial Least Squares Structural Equation Modeling (PLS-SEM). 3rd Edn. Thousand Oaks, CA: SAGE Publications.

[ref24] Heath R. Jayachandran S. (2018). “The causes and consequences of increased female education and labor force participation in developing countries,” in The Oxford Handbook of Women and the Economy, eds. Averett S. L. Argys L. M. Hoffman S. D. (New York: Oxford University Press), 345–367.

[ref25] Hooper D. Coughlan J. Mullen M. R. (2008). Structural equation modelling: guidelines for determining model fit. Electron. J. Bus. Res. Methods 6, 53–60. doi: 10.21427/D7CF7R

[ref26] Hu L. Bentler P. M. (1999). Cutoff criteria for fit indexes in covariance structure analysis: conventional criteria versus new alternatives. Struct. Equ. Model. 6, 1–55. doi: 10.1080/10705519909540118

[ref27] International Labour Organization (2023). World Employment and Social Outlook: Trends 2023. Geneva: ILO.

[ref28] International Labour Organization (2024). Global Employment Trends for Women 2024. Geneva: ILO.

[ref29] Jain T. Chatterjee U. (2024). ICT adoption and female labour supply: evidence from India. World Dev. 174:106406. doi: 10.1016/j.worlddev.2023.106406

[ref30] Jayachandran S. (2021). Social norms as a barrier to women’s employment in developing countries. IMF Econ. Rev. 69, 576–595. doi: 10.1057/s41308-021-00140-w

[ref31] Kabeer N. (1999). Resources, agency, achievements: reflections on the measurement of women’s empowerment. Dev. Change 30, 435–464. doi: 10.1111/1467-7660.00125

[ref32] Kabeer N. (editor). (2001). “Reflections on the measurement of women’s empowerment,” in Discussing Women’s Empowerment: Theory and Practice (SIDA Studies No. 3), (Stockholm: Swedish International Development Cooperation Agency).

[ref33] Kabeer N. (2021). “Gender equality, inclusive growth, and labour markets,” in Women’s Economic Empowerment: Insights from Africa and South Asia, eds. Grantham K. Dowie G. de Haan A. (London: Routledge), 13–48.

[ref34] Kabeer N. Natali L. (2023). Gender equality, labour markets, and social protection. Fem. Econ. 26, 1–26. doi: 10.1080/13545701.2020.1743338, 37339054

[ref35] Kabeer N. Santos F. (2024). Women’s economic empowerment and policy pathways. World Dev. 172:106377.

[ref36] Kerala Startup Mission (2024). Women Startup Ecosystem/Women Programs. Thiruvananthapuram: Government of Kerala.

[ref37] Klasen S. Pieters J. (2022). What explains the stagnation of female labour force participation in developing countries? World Dev. 150:105678. doi: 10.1016/j.worlddev.2021.105678

[ref38] Kocabıçak E. (2025). Women’s labour force participation in developing countries. J. Bus. Soc. Econ. Dev. 3, 12–28.

[ref39] Kudumbashree Mission (2024). Women Entrepreneurship and Livelihood Promotion Report 2024. Thiruvananthapuram: Government of Kerala.

[ref40] Malhotra A. Schuler S. R. Boender C. (2002). Measuring Women’s Empowerment as a Variable in International Development. Washington, DC: World Bank.

[ref41] Mammen K. Paxson C. (2000). Women’s work and economic development. J. Econ. Perspect. 14, 141–164. doi: 10.1257/jep.14.4.141

[ref42] Mehrotra S. Parida J. K. (2022). Why India’s female labour force participation continues to fall. Econ. Polit. Wkly. 57, 45–53.

[ref43] Ministry of Statistics and Programme Implementation (2024). Periodic Labour Force Survey (PLFS) 2023–24 Report. New Delhi: Government of India.

[ref44] Mishra S. Sahoo D. (2025). Impact of women’s reproductive health and empowerment on female labour force participation. J. Labour Mark. Res. 59:Article 24. doi: 10.1186/s12651-025-00414-0

[ref45] Moser C. O. N. (2012). Gender Planning and Development: Theory, Practice and Training. London: Routledge.

[ref46] Press Information Bureau (2024). Female Labour Force Participation Rate Rose to 37% in 2022–23 from 23.3% in 2017–18. New Delhi: Government of India.

[ref47] Ray Hajong V. Kakarlapudi K. K. (2024). Recent Trends in Female Labour Force Participation in Kerala (GIFT Working Paper). Thiruvananthapuram: Gulati Institute of Finance and Taxation.

[ref48] Suri T. Jack W. (2023). Digital financial inclusion and women’s empowerment. Am. Econ. Rev. Pap. Proc. 113, 610–614.

[ref49] Thaddeus K. J. Bih D. Nebong N. M. Ngong C. A. Mongo E. A. Akume D. . (2022). Female labour force participation rate and economic growth in sub-Saharan Africa: “a liability or an asset.”. J. Bus. Soc. Econ. Dev. 2, 34–48. doi: 10.1108/JBSED-09-2021-0118

[ref50] Thomas D. (2022). Intra-household resource allocation and gender inequality. J. Econ. Perspect. 36, 133–156.

[ref51] UN Women (2023). Progress on the Sustainable Development Goals: the Gender Snapshot 2023. New York: UN Women.

[ref52] World Bank (2023). Women, Business and the Law 2023: Removing Barriers to Economic Inclusion. Washington, DC: World Bank Group.

