# Supplementary material for: Women workers’ labour market engagement and financial autonomy: the mediating role of government initiatives in Kerala, India
Source: Front Sociol. 2026 Oct 1;11:1882363. doi: 10.3389/fsoc.2026.1882363 (PMC13668573; doi:10.3389/fsoc.2026.1882363)
Supplement: Supplementary file 1 [file Supplementary_file_1.pdf]

Appendix A: Measurement Instrument

Response Scale: All items were measured using a five-point Likert scale, where 1 = Strongly Disagree, 2 = Disagree, 3 = Neutral, 4 = Agree, and 5 = Strongly Agree.

A1. Cultural Determinants (CULTDET)

Social Norms (CD1–CD4)

CD1. Society encourages women to participate in paid employment.

CD2. Working women are respected in my community.

CD3. Women are encouraged to pursue careers after marriage.

CD4. Community attitudes positively influence women's employment.

Family Role (CD5–CD8)

CD5. My family supports my employment decisions.

CD6. Family members encourage women to become financially independent.

CD7. Household responsibilities are shared among family members.

CD8. Family support motivates women to remain employed.

A2. Economic Determinants (ECONDET)

Economic Factors (ED1–ED4)

ED1. Employment improves household financial security.

ED2. Rising living costs encourage women to work.

ED3. Women's earnings significantly contribute to family income.

ED4. Financial independence motivates women to seek employment.

Employment Opportunities (ED5–ED9)

ED5. Suitable employment opportunities are available for women.

ED6. Local industries provide adequate employment opportunities.

ED7. Employers are willing to recruit women.

ED8. Women receive equal access to job opportunities.

ED9. Skill development improves women's employability.

A3. Cultural Barriers (CB)

Traditional Roles (CB1–CB5)

CB1. Traditional gender roles discourage women from working.

CB2. Marriage limits women's career opportunities.

CB3. Childcare responsibilities reduce women's employment opportunities.

CB4. Social expectations restrict women's career advancement.

CB5. Cultural beliefs discourage women entrepreneurs.

Social Norms (CB6–CB10)

CB6. Society prefers men as primary earners.

CB7. Women experience social pressure to stay at home.

CB8. Women face criticism for pursuing careers.

CB9. Community attitudes discourage entrepreneurship among women.

CB10. Cultural expectations reduce women's workforce participation.

A4. Economic Barriers (EB)

Wage Disparity (EB1–EB3)

EB1. Women receive lower wages than men for similar work.

EB2. Wage inequality discourages women's employment.

EB3. Equal pay opportunities remain limited.

Employment Opportunities (EB4–EB6)

EB4. Women experience difficulty finding suitable employment.

EB5. Limited career opportunities reduce women's participation.

EB6. Promotion opportunities for women are limited.

A5. Institutional Barriers (IB)

Workplace Policy (IB1–IB4)

IB1. Workplace policies adequately support women employees.

IB2. Flexible working arrangements are available.

IB3. Employers provide equal career opportunities.

IB4. Organizations effectively prevent workplace discrimination.

Childcare Facilities (IB5–IB8)

IB5. Affordable childcare facilities are available.

IB6. Workplace childcare support encourages employment.

IB7. Childcare services improve women’s work participation.

IB8. Lack of childcare facilities limits women’s employment.

A6. Labour Market Engagement (LABPART)

LAB1. I actively participate in income-generating activities.

LAB2. I have stable employment or self-employment.

LAB3. My participation in the labour market has increased over time.

LAB4. I intend to continue participating in the labour market.

LAB5. Employment has improved my social status.

A7. Financial Autonomy (FINAUTO)

FIN1. I independently make financial decisions.

FIN2. I control the use of my personal income.

FIN3. I contribute significantly to household financial decisions.

FIN4. I have independent access to financial services.

FIN5. I manage my own savings.

FIN6. I can obtain credit independently.

FIN7. I am financially independent from other family members.

FIN8. My employment has improved my financial security.

FIN9. I feel confident managing my financial resources.

A8. Government Initiatives (GOVINIT)

GOV1. Government programmes encourage women to participate in employment.

GOV2. Government skill development programmes improve employability.

GOV3. Financial assistance schemes support women entrepreneurs.

GOV4. Government policies create employment opportunities for women.

GOV5. Overall, government initiatives effectively promote women’s economic participation.

Table A2: Measurement Sources

Construct	Primary Source
Cultural Determinants	Adapted from the World Values Survey and ILO gender-equality studies
Economic Determinants	Adapted from ILO and World Bank gender statistics frameworks
Cultural Barriers	Adapted from Kabeer (1999) and UN Women frameworks
Economic Barriers	Adapted from ILO gender gap reports
Institutional Barriers	Adapted from the OECD Gender Database and the ILO Decent Work Framework
Labour Market Engagement	Adapted from ILO labour force survey concepts
Financial Autonomy	Kabeer (1999); Malhotra et al. (2002); World Bank women's empowerment framework
Government Initiatives	Adapted from Kudumbashree Mission reports, Ministry of Women and Child Development documents, and the NITI Aayog Women Entrepreneurship Platform

Table A1: Planned District-Wise Allocation of the Target Sample

District	Women Workers	Proportionate Sample	Rounded Sample
Thiruvananthapuram	455,000	378.8	379
Alappuzha	315,000	261.9	262
Ernakulam	470,000	391.1	391
Kozhikode	455,000	378.8	379
Wayanad	108,000	89.9	90
Total	1,803,000	1,500	1,501*

*Source: Census of India (2011) and PLFS (MOSPI, 2024). *The rounded allocation totals 1,501 due to rounding; the final analytical sample after data screening was 1,357.*
